# A heartrending burden of gynaecological cancers in advance stage at nuclear institute of medicine and radiotherapy Jamshoro Sindh

**DOI:** 10.12669/pjms.321.8663

**Published:** 2016

**Authors:** Seema Bibi, Sanober Ashfaque, Naeem Ahmed Laghari

**Affiliations:** 1 Dr. Seema Bibi, MBBS, FCPS, Associate Professor, Department of Obstetrics & Gynaecology, Liaquat University of Medical & Health Sciences, Jamshoro, Sindh, Pakistan; 2 Dr. Sanober Ashfaque, MBBS, DGO, MS, Senior Registrar, Department of Obstetrics & Gynaecology, Liaquat University of Medical & Health Sciences, Jamshoro, Sindh, Pakistan; 3 Dr. Naeem Ahmed Laghari, MBBS, MCPS, DMRT, Director, Nuclear Institute of Medicine & Radiotherapy, Jamshoro, Sindh, Pakistan

**Keywords:** Advance stage, Gynaecological cancer

## Abstract

**Objectives:**

In Pakistan gynaecological cancers are among the leading causes of women’s morbidity and mortality posing huge financial burden on families, communities and state. Due to lack of national cancer registry exact facts and figures are unknown therefore this study was planned to find out prevalence, age, site and stage of presentation of gynaecological cancers at Nuclear Institute of Medicine and Radiotherapy (NIMRA), Jamshoro.

**Methods:**

A retrospective, cross sectional study was conducted from 1^st^ January 2011 to 31^st^ December 2011 at NIMRA Jamshoro. All cases of genital tract cancers were evaluated, required data was entered on predesigned performa and results were analyzed manually.

**Results:**

Out of 2401 total registered cancer cases, 231 (9.6%) patients were suffering from gynaecological cancer making it third most common cancer. Ovary was commonest site followed by cervix and uterus. More than 60% cases presented in advanced stage, mostly during 4^th^ and 5^th^ decade of life.

**Conclusion:**

Gynecological cancer was among top three cancers at one of the busiest public sector cancer institute in Sindh province and significant number presented in advance stage making treatment difficult and expensive. There is urgent need for development and implementation of an effective health policy regarding cancer prevention and treatment.

## INTRODUCTION

Cancers are among the leading cause of morbidity and mortality affecting women worldwide. The burden of caner differs geographically, influenced by various factors like ethnicity, environment, socio-cultural and economic background. One of the distressing trends is the shift of global burden of cancer from developed to underdeveloped countries. Three decades ago, developing countries merely shared 15% of world cancer burden as compared in 2008 where 53% of 12.7 million new cases and 63% of the 7.6 million cancers related deaths occurred in developing countries.^1^,^2^ Gynaecological cancers also follows the same course as 25% of all new cancers occurred in women of developing countries as compared to 16% of developed countries^3^ Poverty, malnutrition, adoption of industrialized life style, and high prevalence of oncogenic viral infections like HPV have been attributed for rise of cancer in resource poor countries.^4^ The misery of cancers is reduced in developed world owing to state owned, well organized health infrastructure having effective screening program leading to early detection and treatment of cancers.

In Pakistan like other resource poor countries, most of women with gynaecological cancers present with advance stage of cancer due to lack of availability and accessibility of cost effective screening, treatment and diagnostic modalities. It is an honest truth that early detection of cancers allows treatment in more curable forms, reduces recovery time and increases the chances of successful treatment. In the context of financial background of Pakistan, the economic impact of early detection of gynecologic cancers is worth to be considering at both micro-economic level of households, firms and macroeconomic level of country’s gross domestic product or its future growth prospects.

Due to lack of national cancer registry in Pakistan, the exact figures about gynaecological malignancies are not known. Therefore to gain acquaintance about problem we decided to collect data from Nuclear Institute of Medicine and Radiotherapy Jamshoro (NIMRA) which is a federally funded institute, established in 1965 at hilly town of Jamshoro, near right bank of river Indus, 15 Km from city of Hyderabad Sindh. It is affiliated with Liaquat University of Medical and Health Sciences Jamshoro/Hyderabad and is equipped with latest technology of nuclear medicine and radiotherapy and has 62 bed indoor facility at Liaquat University Hospital Jamshoro. NIMRA covers wide catchment area, provides both diagnostic and therapeutic facilities to nearly 33,000 patients annually, which are referred from different districts of Sindh province including far flung Tharparkar district.

The study was planned to find out the relative frequency, age distribution and stage of gynaecological malignancies in women attending NIMRA Jamshoro. Results of study will help policy makers, academicians and clinicians in planning future health policies and guidelines for the prevention and management of gynaecological malignancies in special context of Pakistani population.

## METHODS

A one year retrospective cross sectional observational study was conducted from 1^st^ January 2011 to 31^st^ December 2011 at Nuclear Institute of Medicine and Radiotherapy Jamshoro after getting authorization from institute. All women who were registered in NIMRA for diagnosis and/or treatment of genital tract tumors were included. Almost all have histopathologically proven diagnosis. Women suffering from breast or extra genital tract tumours were excluded. Record was evaluated and relevant data was entered on pre-designed performa including age, primary site and stage of presentation according to FIGO classification of tumors. For the purpose of study stage 1 and 2 were considered as early stage cancer while stage 3 and 4 as advance stage carcinoma. Data was analyzed manually and simple frequencies and percentages were drawn.

This study did not require ethics committee approval as study was retrospective, no human intervention was involved and the data provided to the researchers were anonymised. Approval was granted from Director of NIMRA Jamshoro for conducting study in ethical manner.

## RESULTS

During the study period, from 1^st^ January 2011 to 31^st^ December 2011, total 2401 patients were registered at NIMRA Jamshoro with different malignancies. Gynaecological tumors constitute the third most common tumors with frequency of 231(9.62%) proceeded by breast 271(11.28%) and head and neck tumors (31.27%).

The site distribution of gynaecological tumors is shown in Table-I. Ovary was the commonest site, followed by cervix and uterus. Vulva and vagina were rare sites of tumors. Table-II revealed age distribution of study population. Overall the frequency of all tumors increased with age, with 64% of cases developed in women during 4^th^ to 6^th^ decade of life. Ovarian cancers were found in every age group and were the commonest tumor in adolescence girls. Cervical cancers were mostly seen after 30 years of age with peak incidence after fifties. As a matter of fact, gestational trophoblastic tumor is disease of reproductive age group, rarely seen after fifth decade. About 99% of endometrial cancers were seen in menopausal age group. Vulval and vaginal cancers were rare in younger population. Stage of disease is shown in Table-III. More than 60% of all tumors presented in advance stage with the exception of choriocarcinoma cases which are all found in late stages.

**Table-I:** Site distribution of gynaecological tumors N = 231.

S. #	Site of tumor	Number	Percentage
1	Ovary	70	30%
2	Cervix	64	28%
3	Gestational Trophoblastic Tumors	62	27%
4	Endometrium	22	10%
5	Vagina	08	3%
6	Vulva	05	2%

**Table-II:** Age distribution of female genitaltract tumors N = 231.

Age (Year)	Ovarian Cancers	Cervical Cancers	GTN	Endometrial Cancers	Vaginal Cancers	Vulval Cancers	Total No. + %
< 20	5(2%)	0	5(2%)	0	1(0.4%)	0	11(5%)
20–29	8(3%)	3(1%)	26(11%)	0	0	0	37(16%)
30–39	6(3%)	9(4%)	20(9%)	0	0	0	35(15%)
40–49	16(7%)	13(6%)	10(4%)	1(0.4%)	3(1%)	0	43(19%)
50–59	14(6%)	17(7%)	1(0.4%)	10(4%)	2(1%)	3(1%)	47(20%)
> 60	21(9%)	22(10%)	0	11(5%)	2(1%)	2(1%)	58(25%)

**Table-III:** Stage of cancers at the time of presentation N = 175*.

Site	Stage I	Stage II	Stage III	Stage IV	Total

	Early Stage		Advance Stage		
Ovarian Cancer	2(3%)	19(27%)	23(33%)	26(37%)	70(40%)
Cervical Cancer	6(9%)	21(33%)	29(45%)	8(13%)	64(36.5%)
Endometrial Cancer	2(9%)	5(23%)	11(50%)	4(19%)	22(12.5%)
Vaginal Cancer	1(12%)	2(25%)	3(38%)	2(25%)	8(4.5%)
Vulval Cancer	1(20%)	1(20%)	1(20%)	2(40%)	5(3%)
Chorio Carcinoma	0	0	1(17%)	5(83%)	6(3.4%)

* 56 Cases of benign trophoblastic tumors were excluded from this table.

## DISCUSSION

Carcinoma ovary was found to be the most common gynaecological malignancy, in line with other Pakistani studies but contrary to international figures which showed cervical cancer occupying top most position.^5^-^8^ Lack of national cancer registry could be the sole reason behind the disparity. However this difference can also be attributed to Pakistani ethnic and religious background affecting the sexual and reproductive behavior of both male and female population. A woman belonging to this part of world tends to be in monogamous relations and have fewer sexual partners, thus decreasing the chances of oncogenic HPV related infections and cervical cancer. Ritual circumcision of males was also considered as preventive practice but the evidence is not conclusive.

There is close association between early detection of cancers with improved survival and cure rates. When the cancers are spread, treatment becomes difficult, expensive and chances of survival are poor. Regrettably the findings are very much bleak as 60% of all tumors presented at advance stage. The situation is multi-factorial and complex having socio-economic and cultural background along with lack of availability and accessibility of affordable and standardized cancer care in Pakistan, in line with low and middle income countries.^9^,^10^ Illiteracy, poverty and lack of awareness further worsen the situation. Lack of public awareness regarding the benefits of screening, early diagnosis and treatment is a big hurdle in the fight against cancer in resource poor countries. In United States, a research finding concludes that awareness of women and doctors was responsible for dramatic survival for breast cancer before the launch of national screening programme.^11^ In Pakistan, with scarcity of resources and financial constraints, promotion of public awareness is one of low-priced strategy which is worth to try. Mass media including news papers, television, FM radio and cell phones messages can be utilized to promote the importance of early detection and treatment. Lady health workers can be taken on board. It will help women, their families and community to overcome myths, misconceptions and fear regarding cancer.

Ovarian cancer was found to be the commonest tumor of study population, affected women of all age groups ranging from adolescent to post menopausal women and two third of all presented in stage 3 and 4. All over the world ovarian cancers are diagnosed in late stages in-spite of advancement of medical technology and still no effective screening method is recommended. However in developed countries survival rates has improved due to advance surgical techniques, effective chemo-radiotherapy and palliative care.^12^ In Pakistan public sector hospitals which serve majority of population are lacking in surgical expertise and logistic support, radiotherapy centers are scant and chemotherapy is expensive. Therefore patients reach late to concerned hospital and could not complete the treatment timely and properly. There is dire need to develop specialized oncology centers within existing tertiary care hospitals equipped with expert doctors and paramedics, where all surgical, radiotherapy and chemotherapy treatment modalities are available either free or at minimal costs. Primary and secondary care hospitals can be utilized for follow-up to increase compliance. Public private partnership can be considered for developing such centers of excellence, with involvement of philanthropists, taking the example of Sindh Institute of Urology and Transplantation, Karachi.

For prevention of cervical cancer in resource poor countries, the most cost effective screening method is found to be one that requires fewer visits for testing, treatment and follow-up. A holistic approach of simultaneous screening with VIA or VILLI and treatment of pre-cancerous lesions with cryotherapy on same visit is highly effective.^13^ This screen and treat policy needs to be considered for Pakistani population. An integrated health approach utilizing existing health infrastructure and manpower is likely to be beneficial. Doctors and nurses who are working in primary and secondary care settings can be trained for short duration for providing screen and treat services, followed by regular supervision and monitoring. Incorporating HPV vaccination in state owned, expanded program of immunization, targeting adolescent girl population in schools, health care facilities or community settings has the potential to save many lives in resource poor settings.^14^

Prevalence of gestational trophoblastic disease in the study population is in accordance with local and Asian figures.^15^,^16^ The unfortunate finding is presentation of all cases of choriocarcinoma in advance stages, giving insight about the fact that front line health care providers lack knowledge about symptomatology and simple diagnostic modalities for choriocarcinoma which is 100% curative if diagnosed and treated timely. Health education about early symptoms of disease, use of cheap and non invasive easily available diagnostic modalities including serum βhCG, ultrasound and X-ray chest needs to be promoted among health care providers who care women during pregnancy and puerperium.

### Limitation and strength of the study

It includes short duration, lacking minute clinical dates and outcome data. The strength of the study is collection of data from the specialized oncology center having wide catchment area serving thousands of population annually thus giving insight about gravity of situation in population and emphasizes the need for urgent development and implementation of policies by policy makers, stake holders and government to curb the disease.

## CONCLUSION

Gynecological cancer is the third most common cancer among the busy nuclear medicine and radiotherapy center catering thousands of population of Sindh province and majority presented in advance stage thus imposing massive financial burden on state.

## RECOMENDTIONS

Prevention is the gold standard for reducing morbidity and mortality from advance stage gynaecological cancer for country like Pakistan having huge fast growing population, poor resources and very low per capita income. A more practicable way for reducing late diagnosis of cancer is the development and implementation of cost effective prevention strategies including health education, effective vaccination against HPV and utilization of low technology methods for screening and detection of cancer. There is dire need to develop well equipped specialized centers, throughout the country providing affordable treatment to reduce misery of women suffering from advance stage cancer. Political commitment is the key for success.
